# Microcephaly and Associated Risk Factors in Newborns: A Systematic Review and Meta-Analysis Study

**DOI:** 10.3390/tropicalmed7100261

**Published:** 2022-09-24

**Authors:** Natália de L. Melo, Danilo F. de Sousa, Gabriel Z. Laporta

**Affiliations:** 1 Graduate Research and Innovation Program, Centro Universitario FMABC, Santo André 09060-870, Brazil; 2 College of Medicine, Estacio University, Juazeiro 48924-999, Brazil; 3 College of Nursing, Federal University of Ceara, Fortaleza 60020-181, Brazil

**Keywords:** congenital microcephaly, microcephaly, risk factors, health correlates, meta-analysis, population at risk

## Abstract

Congenital microcephaly is caused by a multitude of drivers affecting maternal–fetal health during pregnancy. It is a rare outcome in high-income industrial countries where microcephaly rates are in the range of 0.3–0.9 per 1000 newborns. Prevalence of microcephaly varies considerably across developing countries and can go as high as 58 cases per 1000 live births in pregnancies exposed to infection by Zika virus (ZIKV). Not only ZIKV-infected pregnancies, but other drivers can modulate the occurrence and severity of this outcome. Here, we sought to test the ZIKV–microcephaly association vs. competing hypotheses using a meta-analysis with 8341 microcephaly cases pooled from 10,250,994 newborns in the Americas, Africa, and Asia. Analysis of risk ratios (*RR*) showed teratogens the most likely microcephaly-associated risk factor (*RR* = 3.43; 95%-CI 2.69–4.38; *p*-value < 0.0001), while the statistical significance of the ZIKV–microcephaly association was marginal (*RR* = 2.12; 95%-CI 1.01–4.48; *p*-value = 0.048). Other congenital infections showed strong but variable associations with microcephaly (*RR* = 15.24; 95%-CI 1.74–133.70; *p*-value = 0.014). Microcephaly cases were associated with impoverished socioeconomic settings, but this association was statistically non-significant (*RR* = 2.75; 95%-CI 0.55–13.78; *p*-value = 0.22). The marginal ZIKV–microcephaly association and statistical significance of the competing hypotheses suggest maternal ZIKV infection might not be a cause of microcephaly alone.

## 1. Introduction

Microcephaly is a congenital anomaly of multifactorial origin, possibly related to genetic and chromosomal changes or environmental exposures, including infectious diseases—toxoplasmosis, rubella, cytomegalovirus, and herpes (TORCH)—detrimental substances (alcohol, drugs, radiation), and severe malnutrition [1,2]. The anomaly is characterized by fetal brain subdevelopment, resulting in a lower than standard newborn head circumference. To diagnose a newborn with microcephaly, his or her occipitofrontal circumference must be two standard deviations below the expected mean for gestational age [1,3]. Microcephaly can be severe, present neurological underdevelopment, further imply a life-course dysfunction of brain, and late child growth and development [4].

The risk of microcephaly was not much relevant in the Brazilian epidemiological scenario until recently [1]. According to the publicly available data from the Information System on Live Births (SINASC), the number of microcephaly cases reported in Brazil were 163 cases in 2010, 154 in 2011, 190 in 2012, 183 in 2013, 163 in 2014, 1758 in 2015, 2276 in 2016, 561 in 2017, 453 in 2018, 366 in 2019, and 335 in 2020 [5]. The average number of microcephaly cases in 2010–2014 was 170 per year, then it increased by 10-fold in 2015 (1758 cases), where cases were clustered in the cities of northeastern Brazil, particularly in the state of Pernambuco [6,7]. The World Health Organization declared this event as an emergency of international concern in early 2016 [8]. Following, the Brazilian Ministry of Health supported investigations that proposed an association of microcephaly cases with maternal infection by the Zika virus (ZIKV; Family *Flaviviridae*, Genus *Flavivirus*) [3,6]. ZIKV infections were occurring in humans worldwide after its spread from the African continent with a variety of clinical outcomes [9]. Previously to its spread to the Americas, a ZIKV outbreak unfolded in French Polynesia with eight microcephaly cases identified [10]. Notwithstanding, a few competing hypotheses of underlying causes and mechanisms of congenital microcephaly were further raised and the ZIKV–microcephaly hypothesis started being disputed [11].

The association between maternal ZIKV infection during pregnancy and a microcephaly case was primarily formulated based on spatio-temporal and clinical epidemiological studies [4]. The hypothesis was further supported by studies in biological models showing ZIKV neurotropism towards brain cells [4]. Additionally, the number of microcephaly cases decreased linearly with the decrease in ZIKV incidence reported in pregnant women in Brazil 2016–2020 (Pearson’s correlation = 0.99; *t* = 18.36; *df* = 3, *p*-value = 0.0004). In the Information System for Reporting Diseases (SINAN), the number of reported cases of ZIKV infection in pregnant women in Brazil was 281,464 in 2016, 32,684 in 2017, 19,551 in 2018, 30,500 in 2019, 20,867 in 2020, and 18,680 in 2021 [12,13].

The ZIKV–microcephaly association became disputed by competing hypotheses. One of the reasonings was that microcephaly annually occurred in Brazil with an average of 170 cases per year before the onset of the ZIKV outbreak in 2015 [4]. The competing hypotheses are represented by determinants other than maternal ZIKV infection that can help in triggering microcephaly. Our study arose from the perceived gap in the literature pointing to the need for formal testing of the ZIKV–microcephaly association vs. competing hypotheses (e.g., TORCH). Due to the multifactorial origin of microcephaly and its variable spectrum of exposures, a systematic review and meta-analysis approach was herein applied [14]. The meta-analysis was conducted based on the components of population, exposure, comparator, outcome and study design (PI/ECOS) principle [15]. Patients included were pregnant women and their newborns. Exposures were covariates of microcephaly including maternal ZIKV infection, other congenital infections, use of brain-damaging substances, and socioeconomic factors. Comparison was undertaken by calculating the risk ratio between the risk of microcephaly in the exposed group and the risk of microcephaly in the unexposed group. The outcome was microcephaly. Only observational studies (e.g., cohort studies) were included for further analysis. This study is presented according to the PRISMA recommendation [16] and checklist (Table S1).

The research questions here were the following:

Question 1: Is maternal ZIKV infection associated with the risk of microcephaly?

Question 2: Can exposures other than ZIKV confer further risk to microcephaly?

The research questions’ assumptions were: (1) microcephaly has a multifactorial etiology and (2) the risk of microcephaly increases when pregnant women are exposed to a multitude of associated factors. The study’s impact is to provide decision-makers with basic knowledge for the adoption of preventive measures by public health agencies in Brazil. Finally, this study aims to analyze risk factors associated with microcephaly and assess the ZIKV–microcephaly association vs. competing hypotheses in a specific hypothesis-testing framework.

## 2. Materials and Methods

### 2.1. Study Selection

A literature search of the PubMed, Scopus, and Web of Science databases was carried out from the earliest database date to December 2021. The retrieval method was based on the Medical Subject Headings (MeSH) terms “microcephaly” and “risk factors” plus free words. The search strategy is displayed in Table S2.

### 2.2. Elegibility Criteria

The inclusion criteria were: (1) mothers and their newborns; (2) the case group was exposed to ZIKV, other congenital infections, teratogens, or poor socioeconomic background, and the control group was non-exposed to these risk factors; (3) outcome was microcephaly; and (4) observational studies. The exclusion criteria were: (1) animal or in vitro experiments; (2) studies having low samples sizes; and (3) grey literature.

### 2.3. Data Extraction

The data extracted from the reports and included in the syntheses (qualitative and quantitative) were tabulated according to the information needed for the analytical process and extracted using the Rayyan tool [17]. Among the information observed and recorded for analysis were the following: authorship/year of publication; study design; scenery; participants/sample; exposure; outcome (exposed and non-exposed).

Two reviewers (NLM–first author, DFS–second author) working independently screened each record and each report retrieved to decide whether a study met the eligibility of this review.

### 2.4. Analysed Outcome

The outcome analyzed was microcephaly and its association with covariates as risk factors. Risk factors considered were: (1) socioeconomic background; (2) teratogen; (3) TORCH; and (4) ZIKV. No other outcomes or variables were analyzed.

### 2.5. Quality Assessment

The quality of the selected observational studies was assessed using the Grading of Recommendations Assessment, Development and Evaluation (GRADE) tool [18,19,20] with the following criteria: (1) limitations of study design; (2) inconsistency of the results across studies; (3) precision of the overall estimate across studies; and (4) indirectness of outcome. For observational studies assessed by the GRADE tool, the quality of evidence was higher for cohort studies, but it was lower and linearly decreasing for case–control, cross-sectional, and ecological studies. Inconsistency depended on the statistical heterogeneity among studies in each risk factor group. Precision varied as much as estimates of common effect and random effects model differed to each other. Indirectness of outcome considered microcephaly direct measures in each study. Quality of evidence was then ranked as: high (4 marks), moderate (3 marks), low (2 marks), or very low (1 mark). Finally, funnel plot was used for publication bias analysis.

### 2.6. Summary Measures

Data were imported and analyzed in *R* Software v. 4.0.4 with the *meta* package v. 5.5 [21]. The risk of microcephaly was estimated as a proportion (microcephaly cases/total newborns) per exposure type (exposed to a risk factor, non-exposed). The risk of microcephaly in the exposed group was divided by the risk of microcephaly in the non-exposed group to calculate risk ratio (*RR*) with 95% confidence intervals (95%-CI) in the meta-analysis. A pooled estimate of the risk of microcephaly per risk factors was computed using a random effects model [22] with the *metabin* function [21] and the inverse variance method [23].

Statistical heterogeneity between the studies was assessed using the *Q* and *I*^2^ statistics. The random effects model was used if heterogeneity was high, as indicated by a *p*-value < 0.1 and *I*^2^ ≥ 50%. A common effect model was utilized instead when a low heterogeneity was identified by a *p*-value ≥ 0.1 and *I*^2^ < 50%.

## 3. Results

**Figure 1:**
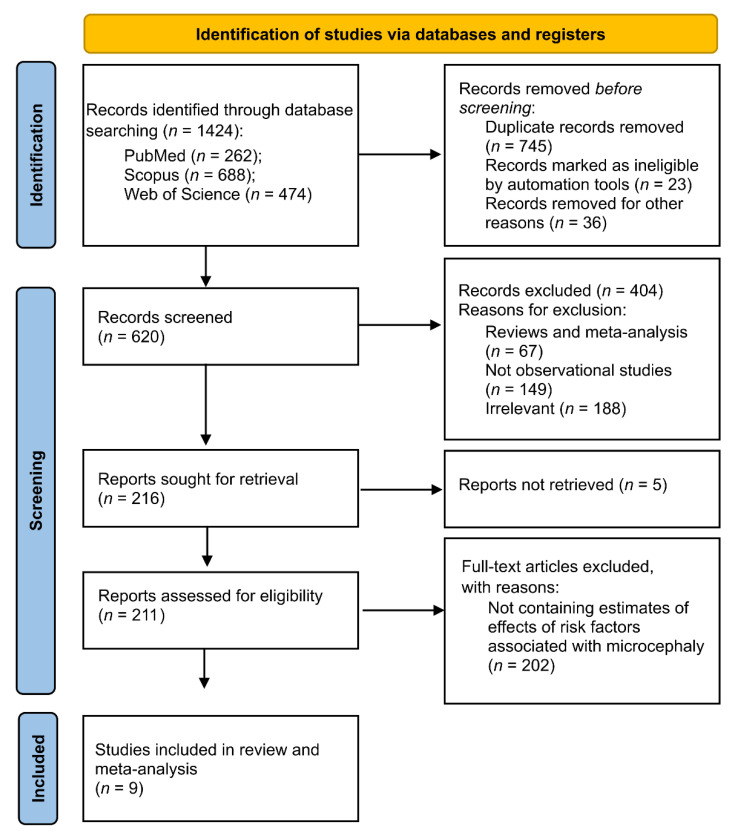
PRISMA 2020 flow diagram for new systematic reviews.

The research found 1424 studies from the three databases PubMed, Scopus, and Web of Science using “microcephaly” and “risk factors” as MeSH terms in the search strategy (Figure 1). After the removal of duplicated and other records, 620 records were screened by two independent researchers (the first and the second authors here). After an initial screening per title and abstract, 404 records were excluded with reasons (Figure 1). Following, a total of 211 studies were retrieved for complete screening and assessed for final eligibility. Out of these, 202 (96%) were excluded because they did not contain estimates of effects from risk factors on microcephaly. Lastly, a total of nine studies were selected and included in review and meta-analysis (Figure 1).

Table 1 summarizes the main information extracted from the selected papers. Five were cohort, two cross-sectional, one case–control, and one ecological study. They showed microcephaly cases associated with risk factors in three continents (the Americas, Africa, and Asia). Newborns were sorted into four groups, as follows: (1) Microcephaly (*n*) Case Group—presented with microcephaly and exposed to risk factor; (2) Newborns Total (*N*) Case Group—exposed to risk factor; (3) Microcephaly (*n*) Control Group—presented with microcephaly and unexposed to risk factor, and (4) Newborns Total (*N*) Control Group—unexposed to risk factor. These data were utilized in the meta-analysis for estimating risk ratio (*RR*) of microcephaly per risk factor. Specific information on data extracted from each study was detailed in the table’s footnotes (Table 1).

**Figure 2:**
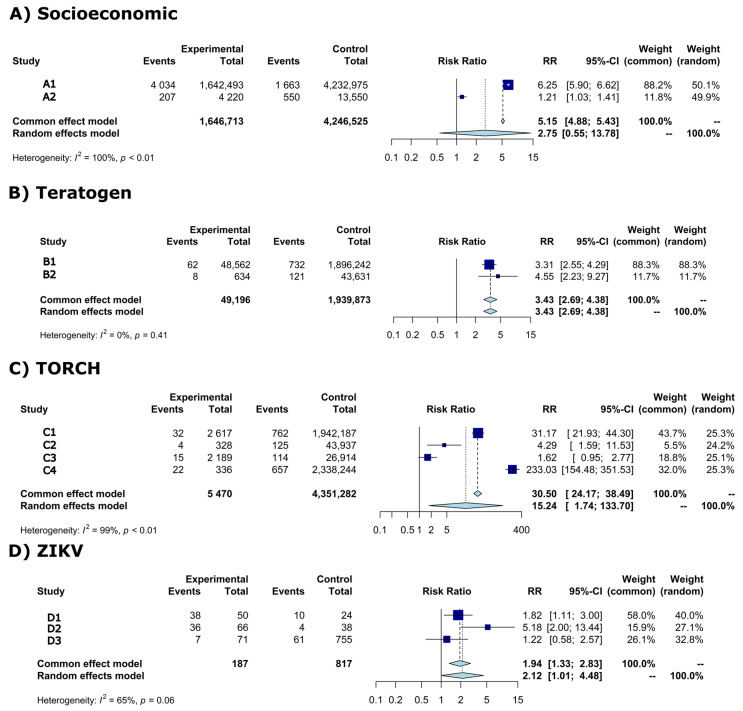
Forest plot of risk ratio (*RR*) and 95% confidence intervals (95%-CI) with light-blue diamonds representing meta-analysis results and dark-blue squares individual study results per risk factor. Vertical solid black line (*RR* = 1) is the risk effect threshold. Dashed black line is the overall microcephaly risk effect from the common effect and random effects model. (**A**) Socioeconomic, (**B**) Teratogen, (**C**) TORCH, (**D**) ZIKV. A1 = Campos et al., 2018 [24]; A2 = Silva et al., 2018 [25]; B1 = Auger et al., 2018 [26]; B2 = Liu et al., 2019 [27]; C1 = Auger et al., 2018 [26]; C2 = Liu et al., 2019 [27]; C3 = Shen et al., 2021 [28]; C4 = Messinger et al., 2020 [29]; D1 = Mendes et al., 2020 [30]; D2 = Robbiani et al., 2019 [31]; D3 = Barsosio et al., 2019 [32].

The meta-analysis showed the risk ratios (*RR*) and CI-95% of microcephaly between exposed (experimental) and non-exposed (control) groups of newborns per risk factor (Figure 2A–D). Individual study results are shown in dark-blue squares and common effect and random effects model are shown in light-blue diamonds (Figure 2A–D).

High statistical heterogeneity was observed in the meta-analysis of socioeconomic, TORCH, and ZIKV risk factors (Figure 2A,C,D)—therefore the random effects model was considered. The random effects model for these risk factors showed the following *RR*s: (1) socioeconomic *RR* = 2.75 (95%-CI: 0.55–13.78; *p*-value = 0.22), (2) TORCH *RR* = 15.24 (95%-CI: 1.74–133.70; *p*-value = 0.014), and (2) ZIKV *RR* = 2.12 (95%-CI: 1.01–4.48; *p*-value = 0.0481).

Low statistical heterogeneity is shown in the meta-analysis of teratogen (Figure 2B), the common effect model was then considered. The common effect model showed a *RR* = 3.43 (95%-CI: 2.69–4.38; *p*-value < 0.0001).

**Figure 3:**
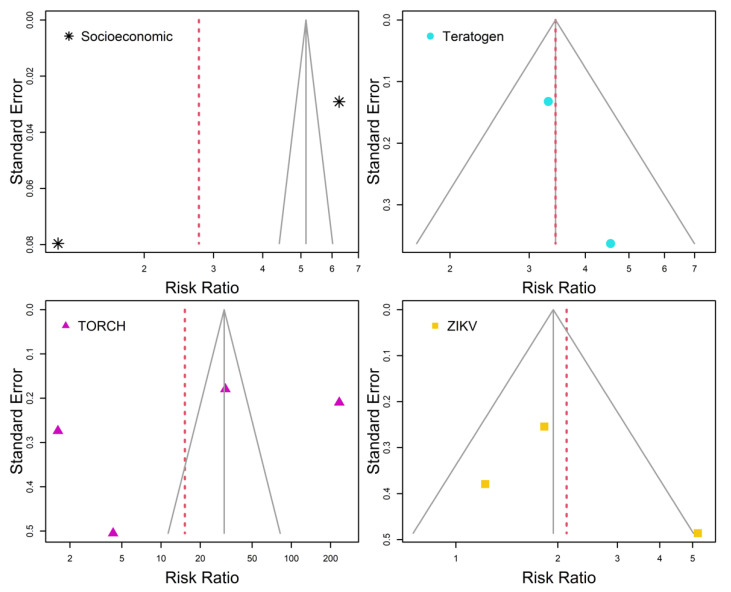
Funnel plots of individual study risk ratio and standard error per risk factors. Solid gray lines are referred to the common effect model. Random effects model estimates are highlighted by dashed red lines. Absence of publication bias occurs when all individual study results are contained within the solid gray lines.

Figure 3 contains funnel plots showing analyses of publication bias among individual study results per risk factor. Each funnel plot (solid gray lines) was depicted in reference to the common effect model for each risk factor. Absence of publication bias was considered when studies were contained inside funnel plots. That was the case in the meta-analysis of studies of microcephaly associated with teratogen (Figure 3–Teratogen). In ZIKV meta-analysis, one of the studies was marginally out of the funnel, thus it can be said that Figure 3–ZIKV shows an absence of publication bias marginally. However, Figure 3–Socioeconomic and Figure 3–TORCH both show publication bias in reference to the association between microcephaly and these risk factors.

Table 2 shows results from quality analysis of selected studies by the GRADE system. These results are convergent to both the forest and funnel plots shown previously. Meta-analyses of socioeconomic and TORCH risk factors were classified as low quality, whereas those for teratogen and ZIKV were of high and moderate qualities, respectively (Table 2).

## 4. Discussion

Our statistical results highlighted associations between risk factors and microcephaly. The quality of evidence was variable among risk factors. The most reliable analysis that with teratogen as a risk factor, followed by analysis of ZIKV, and then TORCH or Socioeconomic. Nevertheless, the effects from any risk factors considered here were reciprocally associated with microcephaly. Stronger or longer exposures to these factors can be linked to a higher or an increased risk of microcephaly. According to this view, the discussion follows with specific interpretations per risk factor.

### 4.1. Socioeconomic Risk Factor

The northeast region of Brazil has the highest poverty rates in the country, with lower socioeconomic indices and poor environmental management [24]. This underdeveloped socioeconomic background is probably responsible for the increase in the *Aedes*. Populations—vectors of dengue and other arboviral diseases [24]. Poverty can also lead to malnutrition and chronic health problems affecting host immunity and the clinical response to infections [25]. Socioeconomic background may not be sufficient to cause microcephaly, but it can be a contributing factor when conjugated with other causes. A previous study showed that the microcephaly–ZIKV outbreak in northeast Brazil 2015–2016 was fueled by poverty added to a concomitant chikungunya outbreak [24].

To better understand the pathogenesis of microcephaly related to poverty, comprehensive large-scale cohort studies carried out in Brazil in 2010, previous to the arrival of ZIKV, were re-analyzed [25]. These authors found that poverty-related variables, including low maternal education, living in monogamy or without a partner, maternal smoking during pregnancy, and preterm and vaginal delivery, were generally more associated with microcephaly [25]. There may be congenital infections closely related to poverty that can trigger microcephaly pathogenesis [25]. It is possible that microcephaly had been endemic to Brazil much before ZIKV circulation.

Immunization rates against infectious diseases are generally high in the Brazilian population [33]. However, vaccination coverage is decreasing lately with a particularly worrying decrease in childhood immunization [34]. This situation may favor infectious disease reemergence, a fact recently observed with the reappearance of measles [35] and yellow fever [36]. As yellow fever vaccine may confer partial protection against ZIKV [37], it is likely the poverty–microcephaly relationship be triggered by settings composed of low vaccine coverage.

### 4.2. Teratogen and TORCH Risk Factors

In Canada and China, maternal exposure to teratogens—alcohol and/or drug abuse—will likely induce fatal underdevelopment [26,27]. From a pooled sample of 1,989,069 newborns with 923 microcephaly cases [26,27], the present study’s analyses showed a microcephaly risk of 343% linked to maternal prenatal and/or perinatal use of substances. These substances are generally consumed in companion with risky behavior, including use of alcohol, tobacco, and other illicit drugs [26,27]. In addition to teratogens, maternal infections including TORCH—toxoplasmosis, rubella, cytomegalovirus, herpes, and others—were determinants of congenital microcephaly in the Chinese birth cohort and in the Canadian retrospective cohort studies [26,27].

Meta-analysis of TORCH showed high heterogeneity and large 95% confidence interval of *RR*. Maternal infection during pregnancy was the strongest risk factor in the Canadian cohort study 1989–2012, with a 32-fold risk of microcephaly compared with no infection, whereas it was the second most important risk factor (after teratogen) in the Chinese birth cohort 2009–2017 [26,27]. The two cities, Quebec and Guangzhou, had no documented evidence of ZIKV infection at the time these studies took place [26,27]. A second study in Guangzhou included 46,610 live births between 2017–2018 and identified 154 microcephaly cases [28]. The authors showed that maternal hepatitis B virus infection, primiparous mothers, preterm labor, and fetal growth restriction were main drivers of microcephaly cases [28]. Although it was the first time that maternal exposure to congenital hepatitis B virus infection has been associated with microcephaly [28], the strength of its effect on microcephaly risk (*RR* = 1.62) is lower than the risks from maternal infection by TORCH (*RR* = 4.29–31.17), as shown by [26,27].

One of the studies that increased variance in the analysis of TORCH was a population-based pooled cohort study of 2,338,580 pregnancies in the USA between 2000–2013 and 2011–2015 [29]. In this study, the authors were interested in testing the association between maternal exposure to cytomegalovirus infection, one of the TORCH diseases, and microcephaly cases in newborns before the circulation of ZIKV [29]. Congenital cytomegalovirus increased microcephaly risk 233-fold (95%-CI: 155–352) [29]. Although the analysis per TORCH risk factor showed high heterogeneity with 95%-CI between 1.7–134, the estimated risk ratio (*RR* = 15) indicates that maternal exposure to any of the TORCH agents (*Toxoplasma gondii*, rubella virus, cytomegalovirus, or herpes simplex virus) or others (hepatitis B virus) during pregnancy will likely result in a greater risk of microcephaly.

It has long been hypothesized that congenital infections beyond TORCH can also contribute to microcephaly [38,39]. Abnormal placental morphologic changes (higher area, perimeter, sprouts, and inflammatory responses) can occur in pregnant women positive for human immunodeficiency virus (HIV) [40]. Maternal HIV infection should not be negligible as a cause of adverse pregnancy outcomes, including microcephaly [41].

### 4.3. ZIKV Risk Factor

Lastly, we compared studies assessing specific effects from ZIKV to microcephaly and obtained two contrasting situations: (1) ZIKV effect as an emerging arboviral disease in Brazil [30,31], (2) ZIKV effect as an endemic arboviral disease in Africa [32]. In the former situation, maternal ZIKV infection during the first trimester of pregnancy was shown as a leading risk factor for the presence of microcephaly [30]. Not only ZIKV-associated infection, but the maternal immune anti-ZIKV responses may contribute to the genesis of microcephaly [31]. In the latter case, microcephaly was shown to be prevalent in Kenya, but it was not ZIKV related [32]. Other drivers, including poverty-related factors and other congenital infections, appeared to be associated with microcephaly [32]. Due to these contrasting situations, the estimate of 95%-CI of the random model risk ratio was 1.01–4.48 (*RR* = 2.12), which means it was statistically significant by a very small margin (1%). This further indicates that ZIKV infection during pregnancy is not acting alone in the causation of microcephaly in newborns.

### 4.4. Limitations

Pooling different types of observational studies in epidemiology (cohort, case–control, cross-sectional, and ecological) can increase heterogeneity in the model estimates due to sampling variation. When heterogeneity was high, our interpretation relied on the random model estimates. Additionally, the use of observational studies for meta-analysis has been encouraged and the selected studies were carried out using standard methodologies [14].

Other databases not included in our analyses, including Embase, have been expanding their coverage in the last years. Notwithstanding, the selected databases (PubMed, Scopus, and Web of Science) can still cover the most comprehensive studies on the topic.

Quality assessment using the GRADE tool is the standard recommendation by the Cochrane Library. The quality of evidence may depend on the type of study design. Randomized trials have higher quality of evidence than observational studies. Here observational studies were used—thus the overall quality of evidence is low. However, in respect to the nature of research questions, the meta-analysis here was the only approach possible.

The recommended number of studies per meta-analysis is 10. Here, meta-analysis was carried out with 2–4 studies. However, the sample sizes analyzed were large: microcephaly *n* = 6454 and newborns N = 5,893,238 in socioeconomic analysis, microcephaly *n* = 923 and newborns N = 1,989,069 in teratogen analysis, microcephaly *n* = 1731 and newborns N = 4,356,752 in TORCH analysis, and microcephaly *n* = 156 and newborns N = 1004 in ZIKV analysis.

## 5. Conclusions

Considering the first research question, we first conclude that maternal ZIKV infection is associated with the risk of microcephaly, but the statistical significance of this association was marginal. In addition, ZIKV infection showed association with microcephaly in Brazil, but not in Africa. Moreover, the ZIKV–microcephaly association in Brazil could have been modulated by other factors (e.g., poverty and/or other congenital infections).

In relation to the second research question, it is highly possible that exposures other than ZIKV confer further risk for microcephaly. Teratogenic factors, including maternal alcohol and/or drug abuse, are the most likely to increase the risk of microcephaly. TORCH showed strong but heterogenous effects on microcephaly, which indicates other modulating factors, such as socioeconomic background, underlying TORCH effects.

It is finally concluded that the risk factors associated with microcephaly in newborns are more likely related to the additive contribution of competing hypotheses (teratogens, TORCH, and socioeconomic background) than ZIKV alone.

## Figures and Tables

**Table 1:** Summary of the extracted data from the 9 studies included in meta-analysis.

Study	Microcephaly (*n*) Case Group	Newborns Total (*N*) Case Group	Microcephaly (*n*) Control Group	Newborns Total (*N*) Control Group	Type of Study	Factor	Sampling Origin
Campos et al., 2018 [24]	4034	1,642,493	1663	4,232,975	Ecological ^1^	Socioeconomic	Brazil
Silva et al., 2018 [25]	207	4220	550	13,550	Cohort ^2^	Socioeconomic	Brazil
Auger et al., 2018 [26]	62	48,562	732	1,896,242	Cohort ^3^	Teratogen	Canada
Liu et al., 2019 [27]	8	634	121	43,631	Cohort ^4^	Teratogen	China
Auger et al., 2018 [26]	32	2617	762	1,942,187	Cohort ^3^	TORCH	Canada
Liu et al., 2019 [27]	4	328	125	43,937	Cohort ^4^	TORCH	China
Shen et al., 2021 [28]	15	2189	114	26,914	Cross-sectional ^5^	TORCH	China
Messinger et al., 2020 [29]	22	336	657	2,338,244	Cohort ^6^	TORCH	US
Mendes et al., 2020 [30]	38	50	10	24	Cohort ^7^	ZIKV	Brazil
Robbiani et al., 2019 [31]	36	66	4	38	Cross-sectional ^8^	ZIKV	Brazil
Barsosio et al., 2019 [32]	2	23	92	945	Case–Control ^9^	ZIKV	Kenya

^1^ Microcephaly cases in Brazil 2015–2016 [5], according to the hypothesis raised by [24] in which the northeast Brazil region is the case group whereas north, southeast, south, and mid-west regions are the control group, ^2^ Microcephaly cases per Brazilian birth cohort studies of 2010, including São Luís cohort study as the case group (lower socioeconomic) and Ribeirão Preto pooled cohort studies as the control group (higher socioeconomic), ^3^ Microcephaly cases in a large cohort study 1989–2012 in Quebec, Canada, per case group (TORCH—Yes, i.e., maternal infections including toxoplasmosis, rubella, cytomegalovirus, and herpes; Teratogen—Yes, i.e., maternal history of alcohol or drug abuse) and control group (TORCH, Teratogen—No), ^4^ Microcephaly cases in a retrospective cohort study 2009–2017 in Guangzhou, China, per case group (TORCH; Teratogen—Yes) and control group (TORCH, Teratogen—No), ^5^ Microcephaly cases in 29,113 live births 2017–2018 in Guangzhou Women and Children’s Medical Centre, China, per case group (Hepatitis B Virus—Yes, i.e., maternal hepatitis B virus carriers) and control group (Hepatitis B Virus—No), ^6^ Microcephaly cases in a pooled population-based cohort study 2000–2013 and 2011–2015 in United States, per case group (congenital cytomegalovirus—Yes, i.e., two or more episodes of cytomegalovirus infection in the infant records between delivery and 90 days after delivery) and control group (congenital cytomegalovirus—No), ^7^ Microcephaly cases at birth in a cohort of children with congenital Zika syndrome born in 2015–2018, Brazilian Maranhão State, per case group (1st trimester of maternal ZIKV infection) and control group (2nd/3rd trimesters of maternal ZIKV infection), ^8^ Microcephaly cases whose mothers had clinical evidence of congenital Zika syndrome and were born at three hospitals in Salvador, Bahia, Brazil, Nov/2015–Feb/2016, per case group (high-to-intermediate levels of anti-ZIKV immune activity in infant and maternal sera) and control group (low levels), ^9^ Microcephaly cases in a nested case–control study of surveillance data from live births in Kenya, 2012–2016, per case group (IgM antibody responses against ZIKV in cord plasma) and control group (no detection).

**Table 2:** Quality analysis of individual-based studies by the GRADE System.

Quality Assessment						Summary of Findings				
Study	Type of Study	Design	Consistency	Directness	Precision	N Patients		Effect		Quality
						Microcephaly	Total	Common	Random	
Campos et al., 2018 [24]	Ecological (1)	Low-Moderate (2.5)	Very Low (1)	High (4)	Very Low (1)	6454	5,893,238	-	*RR* = 2.8 95%-CI = 0.6–13.8 *p*-value = 0.22	⨁⨁⨀⨀ Low (2.1)
Silva et al., 2018 [25]	Cohort (4)									
Auger et al., 2018 [26]	Cohort (4)	High (4)	High (4)	High (4)	High (4)	923	1,989,069	*RR* = 3.4 95%-CI = 2.7–4.4 *p*-value < 0.0001	-	⨁⨁⨁⨁ High (4)
Liu et al., 2019 [27]	Cohort (4)									
Auger et al., 2018 [26]	Cohort (4)	Moderate-High (3. 5)	Very Low (1)	High (4)	Very Low (1)	1731	4,356,752	-	*RR* = 15.2 95%-CI = 1.7–133.7 *p*-value = 0.014	⨁⨁⨀⨀ Low (2.3)
Liu et al., 2019 [27]	Cohort (4)									
Shen et al., 2021 [28]	Cross-sectional (2)									
Messinger et al., 2020 [29]	Cohort (4)									
Mendes et al., 2020 [30]	Cohort (4)	Moderate (3)	Low (2)	High (4)	Moderate (3)	156	1004	-	*RR* = 2.1 95%-CI = 1.01–4.5 *p*-value = 0.048	⨁⨁⨁⨀ Moderate (3)
Robbiani et al., 2019 [31]	Cross-sectional (2)									
Barsosio et al., 2019 [32]	Case-control (3)									

## Data Availability

Not applicable.

## Supplementary Materials

The following supporting information can be downloaded at: https://www.mdpi.com/article/10.3390/tropicalmed7100261/s1, Table S1: PRISMA 2020 Checklist; Table S2: Search strategy.
