# Supplementary material for: Microcephaly and Associated Risk Factors in Newborns: A Systematic Review and Meta-Analysis Study
Source: Trop Med Infect Dis. 2022 Sep 24;7(10):261. doi: 10.3390/tropicalmed7100261 (PMC9611276; doi:10.3390/tropicalmed7100261)
Supplement: Supplementary file 1 [file tropicalmed-07-00261-s001.zip › Table S2 - Search strategy.pdf]

Table S2. Search strategy.

Search number	Search details
#1	"microcephaly" [MeSH Term], "risk factors" [MeSH Term]
#2 PubMed	("microcephaly"[All Fields] OR "congenital microcephaly"[All Fields] OR "severe congenital microcephaly"[All Fields] OR "congenital microcephalies"[All Fields] OR "microcephalies"[All Fields] OR "microencephaly"[All Fields] OR "microlissencephaly"[All Fields] OR "microlissencephalies"[All Fields]) AND ("risk factors"[All Fields] OR "social risk factors"[All Fields] OR "health correlates"[All Fields] OR "population at risk"[All Fields] OR "populations at risk"[All Fields] OR "risk scores"[All Fields] OR "risk score"[All Fields] OR "risk factor scores"[All Fields] OR "risk factor score"[All Fields])
#3 Scopus	TITLE-ABS-KEY("microcephaly" OR "congenital microcephaly" OR "severe congenital microcephaly" OR "congenital microcephalies" OR "microcephalies" OR "microencephaly" OR "microlissencephaly" OR "microlissencephalies") AND TITLE-ABS-KEY("risk factors" OR "social risk factors" OR "health correlates" OR "population at risk" OR "populations at risk" OR "risk scores" OR "risk score" OR "risk factor scores" OR "risk factor score")
#4 Web of Science	ALL=(microcephaly OR congenital microcephaly OR severe congenital microcephaly OR congenital microcephalies OR microcephalies OR microencephaly OR microlissencephaly OR microlissencephalies) AND ALL=(risk factors OR social risk factors OR health correlates OR population at risk OR populations at risk OR risk scores OR risk score OR risk factor scores OR risk factor score)
